# Effects of heat‐killed *Lactiplantibacillus plantarum* TWK10 on exercise performance, fatigue, and muscle growth in healthy male adults

**DOI:** 10.14814/phy2.15835

**Published:** 2023-10-10

**Authors:** Yi‐Chen Cheng, Chia‐Chia Lee, Mon‐Chien Lee, Han‐Yin Hsu, Jin‐Seng Lin, Chi‐Chang Huang, Koichi Watanabe

**Affiliations:** ^1^ Culture Collection & Research Institute, SYNBIO TECH Incorporation Kaohsiung Taiwan; ^2^ Graduate Institute of Sports Science National Taiwan Sport University Taoyuan Taiwan; ^3^ Department of Animal Science and Technology National Taiwan University Taipei Taiwan

**Keywords:** exercise performance, fatigue, heat‐killed, *Lactiplantibacillus plantarum* TWK10, muscle, postbiotic

## Abstract

Consumption of *Lactiplantibacillus plantarum* TWK10 (TWK10) has beneficial probiotic effects, improves exercise endurance performance, regulates body composition, and mitigates aging‐related problems in mice and humans. Here, we investigated the effects of heat‐killed TWK10 on exercise endurance performance, muscle weight and strength, fatigue, and body composition in a double‐blind, placebo‐controlled clinical trial. Thirty healthy males aged 20–40 years were assigned to the Control group or heat‐killed TWK10 group (TWK10‐HK) in a balanced order according to each individual's initial maximal oxygen uptake. After 6‐week administration, the exercise endurance time in the TWK10‐HK was significantly increased (*p* = 0.0028) compared with that in the Control group. The grip strength on the right and left hands of the subjects was significantly increased (*p* = 0.0002 and *p* = 0.0140, respectively) in the TWK10‐HK compared with that in the Control group. Administration of heat‐killed TWK10 resulted in a significant increase (*p* = 0.0275) in muscle weight. After 6‐week administration, serum lactate, and ammonia levels were significantly lower in the TWK10‐HK group than in the Control group during the exercise and recovery periods. These findings demonstrate that heat‐killed TWK10 has significant potential to be used as a postbiotic for humans.

## 1 INTRODUCTION

Probiotics are defined as “live microorganisms that, when administered in adequate amounts, confer a health benefit on the host”. The key health benefits of probiotics include altering the gut microbial composition, improving intestinal health, and immune system homeostasis. Probiotics act as co‐adjuvants, and they may reduce the risk of and be used to treat gastrointestinal and immune‐associated diseases (Hill et al., 2014). Probiotics are used as potential ergogenic aids to enhance exercise capacity (Marttinen et al., 2020). Administration of probiotics has been shown to increase the time required for fatigue in animals, athletes, and non‐athletes in clinical studies (Chen et al., 2016; Huang et al., 2019; Shing et al., 2014; Soares et al., 2019). Recent studies have shown that consumption of non‐viable microorganisms, such as heat‐killed (or heat‐inactivated) probiotics (postbiotics), cell‐free supernatants, or purified key components, has advantageous effects, specifically immune‐modulating effects including protection against enteropathogens and maintenance of gut barrier integrity (Biswas et al., 2013; Li et al., 2009; Salminen et al., 2021; Sang et al., 2013; Ueno et al., 2011). Additionally, the efficacy of postbiotics in reducing fatigue and increasing muscle weight has been demonstrated. Supplementation with heat‐killed *Lactococcus lactis* JCM 5805 for 13 days in male athletes engaged in high‐intensity exercise (>11 h per week) modulated autonomic nerve functions, resulting in a decrease in fatigue via the regulation of toll‐like receptor 9 (Komano et al., 2018). Supplementation with heat‐killed *Lactobacillus gasseri* CP2305 in long‐distance relay race runners for 12 weeks, significantly facilitated recovery from fatigue, and relieved anxiety and depressive mood (Sawada et al., 2019). Heat‐killed *Bifidobacterium breve* B‐3 administration for 4 weeks increased muscle weight and modified metabolic functions, possibly through the Akt, and AMPK pathways in rats (Toda et al., 2020).

Viable *Lactiplantibacillus plantarum* TWK10 (TWK10) has been demonstrated to improve endurance performance (time‐to‐exhaustion, TTE) and to increase muscle weight and strength in both mice and humans (Chen et al., 2016; Huang et al., 2018 and 2019). Furthermore, administration of high‐dose heat‐killed TWK10 (3 × 10^11^ cells/day) has been demonstrated to enhance exercise endurance performance and to reduce fatigue and responses to exercise‐induced inflammation in humans (Lee et al., 2022). In this study, we reduced the administration dose of heat‐killed TWK10 and investigated the effects on exercise performance, muscle strength, and body composition in a placebo‐controlled, double‐blinded clinical trial.

## 2 MATERIALS AND METHODS

### 2.1 Preparation of *L. plantarum* TWK10

TWK10 was isolated from Taiwanese pickled cabbage, “Pao‐tsai” (Chen et al., 2013), and identified as *Lactiplantibacillus plantarum* subsp. *plantarum* (Hsu et al., 2022). Heat‐killed TWK10 cells were prepared by SYNBIO TECH INC. (Kaohsiung, Taiwan) in capsule form with a dose of 1.5 × 10^10^ cells (corresponding to 1.5 × 10^10^ colony‐forming units) standardized with microcrystalline cellulose and maltodextrin. Heat‐killed TWK10 cells were prepared by heating the liquid bacterial culture at 70°C for 60 min followed by centrifugation to collect bacterial cell pellets and spray‐drying pellets for capsule preparation. The ingredients of the placebo capsule were the same as those of the heat‐killed TWK10 capsule except for the omission of TWK10. The absence of viable bacteria in the heat‐killed TWK10 and placebo capsules was confirmed by plating the contents of the capsules on de Man, Rogosa, and Sharp (MRS; BD Difco) agar plates followed by anaerobic incubation at 37°C for 48 h.

### 2.2 Subjects

Thirty healthy male adults (aged 20–40 years) without professional exercise training were recruited in this study. Subjects were excluded from this study if they had heart/cardiopulmonary disease, diabetes, thyroid‐related disease, hypogonadism, hepatorenal disease, musculoskeletal disorder, neuromuscular/neurological disease, autoimmune disease, cancer, peptic ulcers, ulcerative colitis, Crohn's disease, or a body mass index (BMI) > 27. During the experimental period, all participants were prohibited from taking probiotics, prebiotics, fermented products (yogurt or other foods), vitamins, minerals, herbal extracts, dietary supplements for exercise and athletic performance, and antibiotics to avoid their interference. Subjects who agreed to follow the study protocol and voluntarily signed an informed consent form were included in this study. The study was reviewed and approved by the Institutional Review Board of Landseed International Hospital (Taoyuan, Taiwan; LSHIRB No. 19‐027‐A2). The subjects were recommended to maintain their usual lifestyle, physical activities, and diet.

### 2.3 Experimental design

**FIGURE 1:**
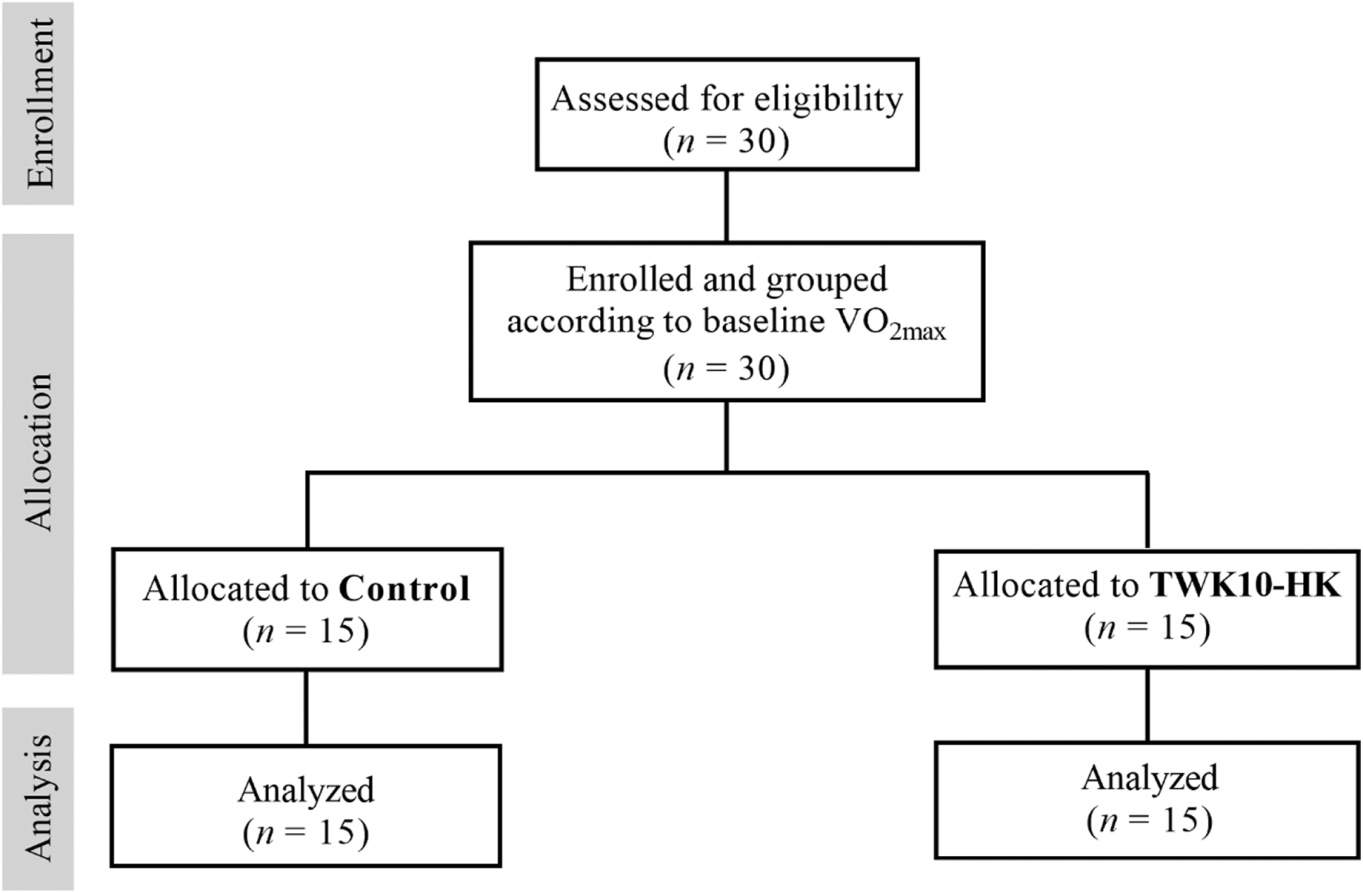
CONSORT flow diagram of this study.

This was a double‐blind, placebo‐controlled trial, with a 2‐week wash‐out period, and a 6‐week intervention period. Before the trial, body height, body weight (BW), BMI, body composition, blood biochemical parameters, and exercise endurance capacity of the participants were measured. The subjects were assigned to Control (placebo, *n* = 15) or TWK10‐HK (heat‐killed TWK10, 3 × 10^10^ cells/day, *n* = 15) groups in a balanced order based on their basal maximal oxygen consumption (VO_2max_). After 2 weeks of wash‐out period, the subjects were required to take two placebos or TWK10‐HK capsules daily after meal for 6 weeks, and then assessed for exercise endurance performance, grip strength, fatigue‐related parameters, and body composition. Dietary information of the subjects was recorded before and after administration, prior to the exercise endurance performance test. The intake of carbohydrates, proteins, fats, and total calories was analyzed by professional nutritionists as reference values. A description of the experimental procedure is shown in Figure 1.

### 2.4 VO_2max_ and endurance performance tests

The maximum oxygen consumption and exercise performance of the subjects were evaluated using a treadmill (Pulsar, h/p/cosmos) and an automatic breathing analyzer (Vmax 29c, Sensor Medics). A polar heart rate device was used to monitor the heart rate (HR). According to Bruce's protocol, the treadmill speed started at 7.2 km/h and was increased by 1.8 km/h every 2 min until subjects experienced fatigue (Bruce et al., 1973). The maximum oxygen consumption was considered when the respiratory exchange rate (the volume ratio of carbon dioxide produced to oxygen consumed, VCO_2_/VO_2_) was higher than 1.10 and reached the maximum HR (maximum HR = 220 − age). The three highest VO_2max_ peaks were averaged to obtain the VO_2max_ values of individual subjects. The individual basal VO_2max_ during the pre‐test was used as a reference to adjust the appropriate exercise intensity individually to measure physiological adaptation (60% VO_2max_) and exhaustive endurance (85% VO_2max_). The subjects performed the maximum endurance test using a treadmill, warmed up at 60% VO_2max_ intensity for 5 min, and then started the endurance running test at an 85% VO_2max_ workload. To assess the maximum exercise tolerance of the subjects, we monitored their physical conditions every 5 min using HR and Borg's rating of perceived exertion (RPE) and continued to record until subjects experienced exhaustion. The sustained exercise duration was recorded as an endurance index. The detailed formula for intensity adjustment was based on that used in a previous study (Huang et al., 2018).

### 2.5 Handgrip strength test

Handgrip strength was measured in kg using a Takei digital grip strength meter (T. K. K. 5401, Takei Scientific Instruments Co., Ltd.). Before the formal test, the subjects were required to squeeze the gripper with minimal force to confirm their understanding of the operating procedure and the gripping distance. The researchers randomly designated the dominant or non‐dominant hand to start the test. During the test, the subjects were asked to squeeze the gripper with one hand with maximum effort, maintain squeezing for at least 5 s, and repeat the test by changing hands at 60 s intervals to prevent fatigue. The subjects repeated the exchange method three times, and the individual maximum grip strengths of the two hands were recorded (Yang et al., 2018).

### 2.6 Fatigue‐associated biochemical indices and hematology profiling

To assess fatigue‐related indicators, subjects fasted for at least 8 h before the fixed intensity exercise challenge (FIEC) (60% VO_2max_). Blood samples were collected using an arm venous catheter at the indicated time points during the exercise and recovery periods, including baseline (0), 15 (E15), and 30 (E30) min during the exercise phase, and 20 (R20) and 60 (R60) min in the recovery phase. Serum levels of lactate, ammonia, glucose, and creatinine kinase (CK) were assessed to monitor physiological adaptation. All biochemical indicators were determined using an autoanalyzer (Hitachi 7060). Complete blood count (CBC) profiles (Mindray BC‐2800Vet) were also determined at the 60‐min time point in the recovery phase.

### 2.7 Body composition

Body composition was measured before the trial and FIEC using a bioelectrical impedance analyzer (BIA) with an InBody 570 device (In‐body, Seoul, South Korea) after an 8 h fast. The device was designed using a multi‐frequency principle and provided 1, 5, 50, 260, 500, and 1000 kHz frequency screening in 60 s. For measurement, the subjects stood on the foothold electrodes after the subjects' palms and soles were cleared; they held the sensing handle using both hands, and the subjects kept their arms open and away from their body at a 30° angle without speaking and moving during the measurement period.

### 2.8 Principal component analysis

To evaluate the impact of the administration of heat‐killed TWK10 on the physical features of the subjects, that is, 30 subjects in the Control and TWK10‐HK groups were analyzed before and after administration on the basis of seven physical features (BW, BMI, muscle weight, body fat mass, grip strength [right and left hands], and endurance time) using principal component analysis (PCA). GraphPad Prism 9.3.1 (GraphPad) was used to generate PCA plots using the first two principal components according to their group.

### 2.9 Statistical analysis

**FIGURE 2:**
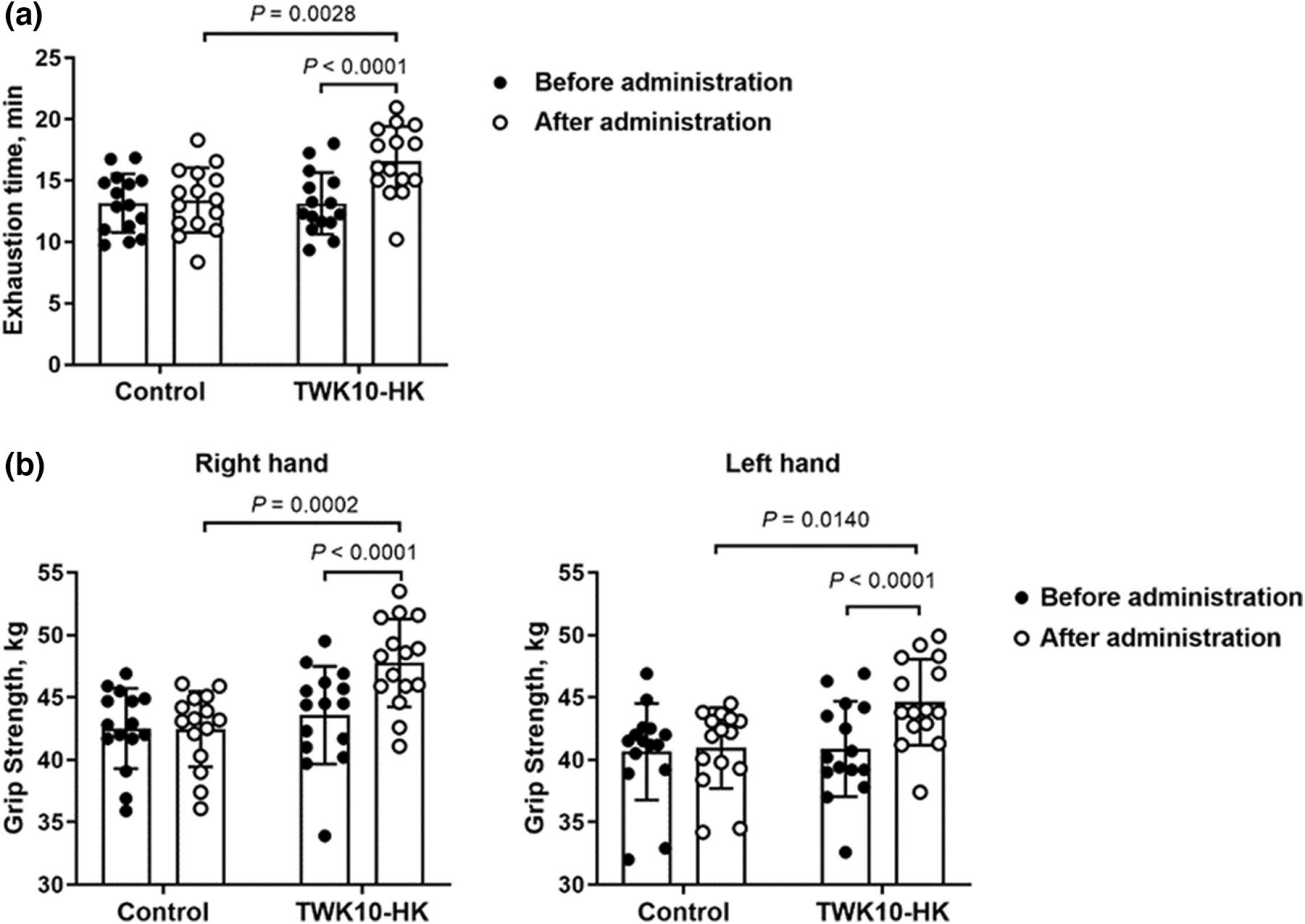
Effects of heat‐killed TWK10 administration on (a) exercise endurance performance and (b) grip strength of the subjects. Data are shown as mean ± SD. The statistical significance was analyzed by two‐way repeated measures ANOVA with Bonferroni *post‐hoc* test.

All data are expressed as the mean ± SD. Statistical analyses were performed using GraphPad Prism 8.1.1 (GraphPad). Within‐group differences (before administration vs. after administration) were analyzed using the paired Student's *t*‐test for parametric comparisons, and the Wilcoxon signed‐rank test was used for non‐parametric paired comparisons, including those for BMI and fat mass. Intergroup differences (Control group vs. TWK10‐HK group) were analyzed using the unpaired Student's *t*‐test for parametric comparison, and the Mann–Whitney *U*‐test was used for non‐parametric comparisons, including the neutrophil, lymphocyte, monocyte, eosinophil, basophil, mean corpuscular hemoglobin concentration (MCHC), the ratio of the neutrophil count to the lymphocyte count (NLR), the ratio of platelets to lymphocytes (PLR), BMI, and fat mass. The statistical significance of exercise endurance performance and grip strength (Figure 2) were analyzed using two‐way repeated measures ANOVA with Bonferroni *post‐hoc* test. Differences were considered statistically significant at *p* < 0.05.

## 3 RESULTS

### 3.1 Effects of heat‐killed TWK10 on endurance performance and handgrip strength

The basic demographic profiles and characteristics of the subjects are presented in Table 1. The basal level of endurance performance of the subjects was evaluated using the TTE test with 85% VO_2max_ workload, and no significant difference was observed between the Control and TWK10‐HK groups. After 6 weeks of administration, the exhaustion time in the TWK10‐HK group was significantly increased by 1.24‐fold (*p* = 0.0028) compared to that in the Control group. Furthermore, after 6 weeks of administration, the mean exhaustion time in the TWK10‐HK group was significantly increased by 1.26‐fold (*p* < 0.0001) compared with the baseline values of subjects, whereas no significant increase was observed in the Control group (Figure 2a).

**TABLE 1:** General characteristics of the subjects.

	Control	TWK10‐HK
	*n* = 15	*n* = 15
Age (years)	20.5 ± 1.5	20.9 ± 2.4
Height (cm)	172.5 ± 5.3	172.4 ± 4.6
Weight (kg)	69.3 ± 7.7	70.5 ± 9.5
VO_2max_ (mL/kg/min)	53.5 ± 8.1	53.4 ± 5.6

*Note*: Subjects were randomly assigned to the control and TWK10‐HK groups. Data are shown as mean ± SD, and intergroup differences were statistically analyzed using unpaired Student's *t*‐test.

In addition to the TTE test, the handgrip strength was measured to assess muscle strength. Before administration of placebo or heat‐killed TWK10 capsules, no significant differences in grip strength were observed between the Control and TWK10‐HK groups in either the right or the left hands of subjects. After 6 weeks of administration, grip strength in the TWK10‐HK group was significantly increased by 1.12‐fold (*p* = 0.0002) and 1.09‐fold (*p* = 0.0140) in the right and left hands, respectively, compared to that seen in the Control group. When compared with baseline values in test subjects, grip strength in the TWK10‐HK group significantly increased after 6 weeks of administration (right hand, *p* < 0.0001; left hand, *p* < 0.0001) (Figure 2b).

### 3.2 Effects of heat‐killed TWK10 on physiological adaptation

**FIGURE 3:**
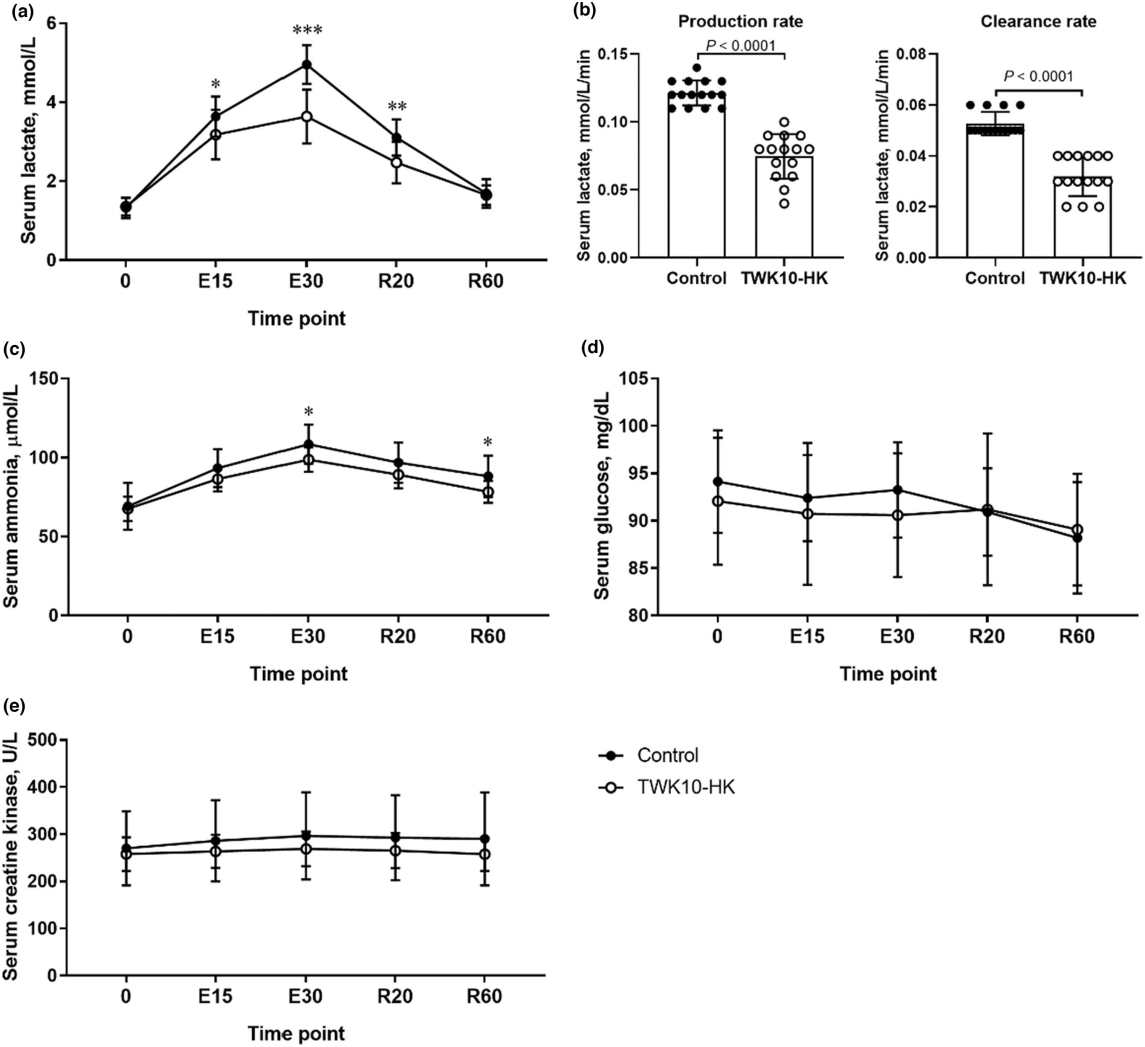
Effects of heat‐killed TWK10 administration on circulating (a, b) lactate, (c) ammonia, (d) glucose, and (e) creatine kinase levels during and after the exercise challenge. Data are shown as mean ± SD. Treatment effects of serum lactate, ammonia, glucose, and creatine kinase levels were statistically analyzed using the unpaired Student's *t*‐test. The production rate and clearance rate of serum lactate were statistically analyzed using Mann–Whitney *U‐*test. Production rate = [(lactate concentration at E30)–(lactate concentration at baseline)]/(exercise duration, 30 min). Clearance rate = [(lactate concentration at E30)–(lactate concentration at R60)]/(recovery period, 60 min). *p < 0.05, **p < 0.01, ***p < 0.001.

Next, fatigue‐related indices were assessed based on the fixed‐intensity exercise challenge with 60% VO_2max_ workload after 6 weeks of administration of placebo or TWK10‐HK capsules. Serum lactate levels increased during the exercise challenge, reaching the maximum after 30 min of the exercise challenge, and gradually decreased to basal levels during the recovery period. A significant reduction in lactate accumulation in the exercise phase (time point: E15 [*p* = 0.0371], E30 [*p* < 0.0001]), and recovery phase (time point: R20 [*p* = 0.0015]), respectively, was observed in the TWK10‐HK group compared with those of the Control group (Figure 3a). Compared with the Control group, the production of serum lactate during the 30‐min exercise challenge and the clearance of serum lactate during the 60‐min recovery phase were significantly decreased in the TWK10‐HK group (*p* < 0.0001) (Figure 3b). The level of serum ammonia in the TWK10‐HK group was significantly reduced during the exercise challenge (time point E30 [*p* = 0.0153]) and recovery period (time point R60 [*p* = 0.0152]) when compared with that in the Control group (Figure 3c). There were no significant differences in glucose (Figure 3d) and creatine kinase (Figure 3e) levels between the Control and TWK10‐HK groups at each collection time point.

### 3.3 Effects of heat‐killed TWK10 on hematology

Blood samples of subjects were collected and analyzed at R60 (the 60‐min time point during the recovery period immediately after the 30 min exercise challenge [60% VO_2max_]) The CBC profiles of the subjects before and after administration were obtained, and NLR and PLR were analyzed. There were no significant differences in WBC, neutrophil, lymphocyte, monocyte, eosinophil, basophil, platelet, and red blood cell (RBC) counts, hemoglobin (Hb) levels, hematocrit (Hct), mean corpuscular volume (MCV), mean corpuscular hemoglobin (MCH), and MCHC levels between or within groups. NLR and PLR, used as markers of exercise‐induced systemic inflammatory responses (Walzik et al., 2021), no significant changes in these ratios were observed in subjects receiving heat‐killed TWK10 compared with those in the Control group (Table 2).

**TABLE 2:** Blood count profiles of subjects.

	Control		TWK10‐HK	
	Before	After	Before	After
WBC (cumm)	6458.67 ± 1171.08	6298.67 ± 846.73	6408.67 ± 1528.45	6226.00 ± 880.46
Neutrophil (%)	58.35 ± 4.80	61.75 ± 6.78	58.41 ± 3.18	61.17 ± 5.51
Lymphocyte (%)	32.16 ± 4.69	28.97 ± 6.52	32.11 ± 3.55	29.32 ± 5.74
Monocyte (%)	5.61 ± 1.20	5.71 ± 1.48	5.87 ± 1.60	6.15 ± 1.47
Eosinophil (%)	2.87 ± 1.11	2.93 ± 1.53	2.97 ± 1.37	2.68 ± 2.65
Basophil (%)	0.74 ± 0.32	0.64 ± 0.23	0.65 ± 0.26	0.68 ± 0.30
RBC (MIL/cumm)	5.13 ± 0.31	5.17 ± 0.36	5.14 ± 0.39	5.20 ± 0.31
Hemoglobin (gm/dL)	15.47 ± 0.86	15.43 ± 0.97	15.49 ± 0.76	15.67 ± 0.92
Hematocrit (%)	45.49 ± 1.33	44.82 ± 2.55	45.37 ± 2.31	45.55 ± 2.04
MCV (fl)	87.27 ± 2.39	86.75 ± 2.86	87.33 ± 2.49	87.75 ± 2.88
MCH (pg)	30.42 ± 1.00	29.86 ± 1.00	29.82 ± 1.49	30.18 ± 1.19
MCHC (%)	34.28 ± 1.18	34.43 ± 0.72	34.45 ± 0.77	34.39 ± 1.08
Platelet (1000/cumm)	273.53 ± 49.62	272.00 ± 52.76	271.13 ± 52.59	271.33 ± 53.61
NLR	1.87 ± 0.40	2.26 ± 0.64	1.85 ± 0.31	2.20 ± 0.62
PLR	136.84 ± 36.55	157.07 ± 44.19	142.10 ± 51.00	153.76 ± 34.19

*Note*: Data are shown as mean ± SD. Intergroup differences were statistically analyzed using the unpaired Student's *t*‐test. Statistical differences in the neutrophil, lymphocyte, monocyte, eosinophil, and basophil counts, MCHC, ratio of the neutrophil count to the lymphocyte count (NLR) and ratio of platelets to lymphocytes (PLR) were analyzed using Mann–Whitney *U*‐test.

### 3.4 Effects of heat‐killed TWK10 on body composition

To evaluate the impact of heat‐killed TWK10 on body composition, BW, BMI, muscle weight, and body fat mass were monitored pre‐ and post‐administration. After 6 weeks of administration, no significant differences in BW, BMI, and body fat mass were observed between the Control and TWK10‐HK groups. The muscle weight in the subjects receiving heat‐killed TWK10 was significantly increased (*p* = 0.0275) compared with their baseline values (Table 3).

**TABLE 3:** Body composition of subjects.

	Control		TWK10‐HK	
	Before	After	Before	After
Body weight (kg)	69.33 ± 7.69	69.15 ± 8.95	70.45 ± 9.53	70.62 ±9.94
BMI (kg/m^2^)	22.94 ± 2.27	23.13 ± 2.44	23.50 ± 2.58	23.55 ± 2.77
Muscle weight (kg)	33.40 ± 2.67	32.73 ± 3.06	33.77 ± 3.33	34.13 ± 3.50*
Fat Mass (%)	15.90 ± 4.84	16.13 ± 5.44	15.73 ± 4.53	15.19 ± 5.27

*Note*: Data are shown as mean ± SD. Effects of heat‐killed TWK10 treatment on body weight and muscle weight were statistically analyzed using the unpaired Student's *t*‐test, and the Mann–Whitney *U*‐test was performed for statistical comparison of BMI and fat mass. Differences on body weight and muscle weight between before and after administration for each group were statistically analyzed using the paired Student's *t*‐test, and the Wilcoxon signed‐rank test was used for statistical comparison of BMI and fat mass.

Abbreviation: BMI, body mass index.

* *p* < 0.05.

### 3.5 Impact of heat‐killed TWK10 on the physical features of subjects

**FIGURE 4:**
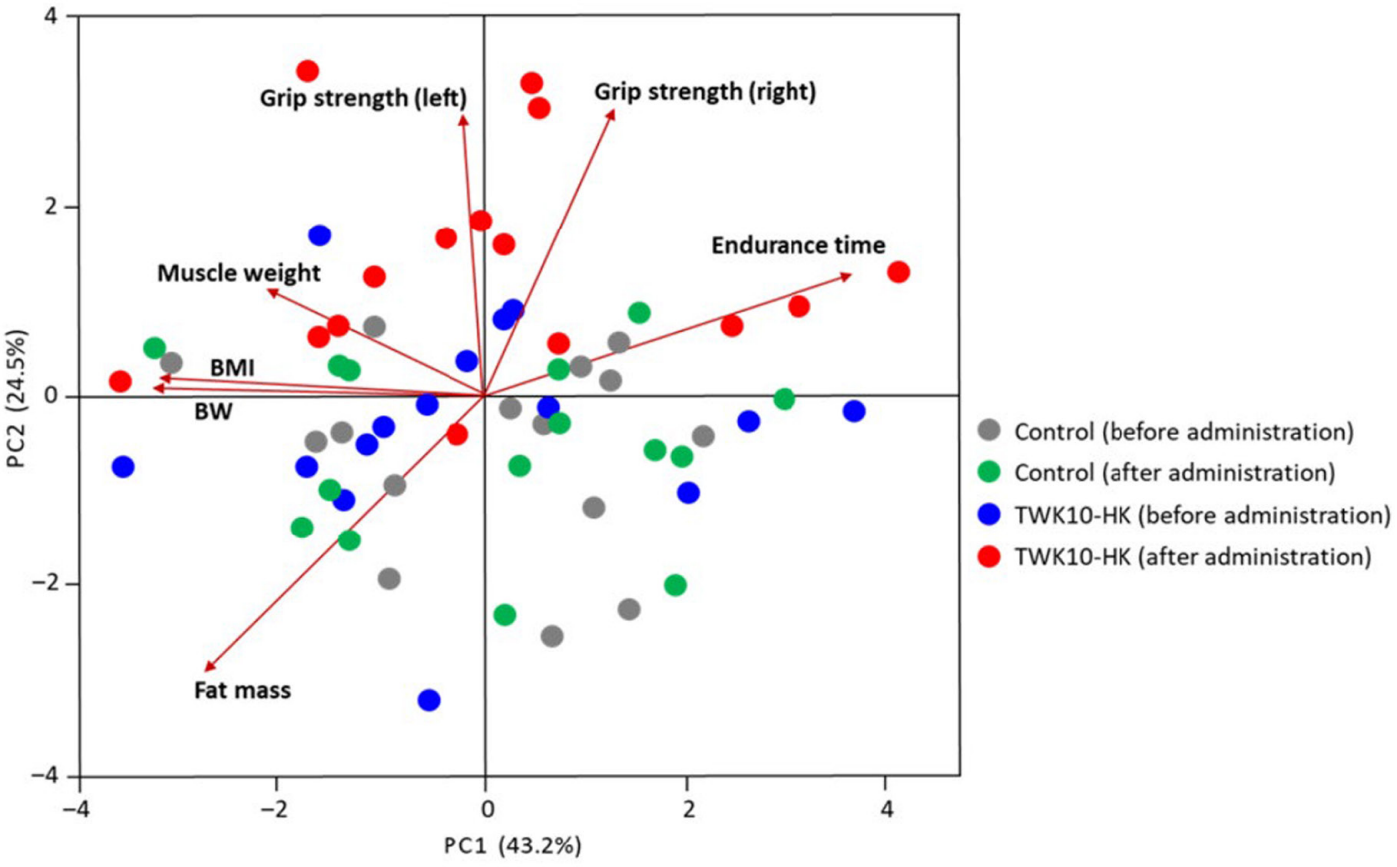
Principal component analysis (PCA) of 30 healthy adults. The data of physical features (BW, BMI, muscle weight, fat mass, grip strength [right and left hands], and endurance time) of 30 subjects were subjected to PCA, and the first two principal components, PC1 and PC2 were plotted. The seven loadings of variables are indicated by arrows together with their variable names.

Using PCA, we decomposed the data of physical features (BW, BMI, muscle weight, fat mass, grip strength [right and left hands], and endurance time) of 30 subjects into two factors that explained 67.7% of the variance. Principal component 1 (PC1) was heavily loaded with grip strength (right hand) and endurance time and negatively loaded with muscle weight, BW, BMI, and fat mass. Principal component 2 (PC2) was heavily loaded with grip strength (both right and left hands), muscle weight, and endurance time and negatively loaded with fat mass. The subjects in the TWK10‐HK group after administration were strongly influenced by grip strength (right and left hands), muscle weight, and endurance time. The subjects in the TWK10‐HK group after administration were clearly differentiated from those before administration (Figure 4).

### 3.6 Dietary information

Dietary details of the subjects were monitored before and after the administration of the placebo and heat‐killed TWK10 capsules. There was no significant difference in the calorific intake between the Control and TWK10‐HK groups (Table 4).

**TABLE 4:** Dietary information of the subjects.

	Control		TWK10‐HK	
	Before	After	Before	After
Carbohydrate (g/day)	221 ± 39	227 ± 43	222 ± 31	226 ± 27
Protein (g/day)	83 ± 14	82 ± 15	82 ± 13	84 ± 10
Lipid (g/day)	88 ± 21	90 ± 21	88 ± 17	92 ± 19
Calorie (kcal/day)	2007 ± 322	2048 ± 328	2009 ± 214	2062 ± 220

*Note*: Data are shown as mean ± SD, and intergroup differences were statistically analyzed using the unpaired Student's *t*‐test.

## 4 DISCUSSION

Previously, we have evaluated the effects of viable and heat‐killed TWK10 on exercise performance, fatigue, and body composition in healthy adults and the elderly. Healthy adults supplemented different doses (9 × 10^10^ and 3 × 10^11^ CFU/day) of viable TWK10 or heat‐killed TWK10 (3 × 10^11^ cells/day) for 6 weeks improved exercise performance and decreased serum lactate and ammonia. Administration of viable TWK10 decreased change of fat mass and increased change of muscle weight (Huang et al., 2019; Lee et al., 2022). Elderly supplemented viable TWK10 (6 × 10^10^ CFU/day) for 18 weeks also increased muscle mass and improved muscle strength (Lee, Tu, et al., 2021). In this study, untrained and healthy volunteers were recruited to evaluate the effect of heat‐killed TWK10 on exercise capacity, muscle strength, physiological adaptation, and body composition. We confirmed that the physical indices, hand grip strength, muscle weight, and endurance time, which are correlated with exercise performance, were remarkably improved by administration of heat‐killed TWK10 for 6 weeks at 1/10th of the dose (3 × 10^10^ cells/day) used in our previous trial. Exercise, a stressor to the body, can induce inflammatory responses through the production of pro‐inflammatory cytokines and reactive oxygen species from activated leukocytes, causing muscle damage and tissue injury (Cerqueira et al., 2020; Silveira et al., 2016; Suzuki, 2018, 2019a, 2019b). In our previous study, the NLR and PLR values, which are used as markers of exercise‐induced systemic inflammatory responses (Walzik et al., 2021), were significantly decreased in subjects receiving heat‐killed TWK10 at a dose of 3 × 10^11^ cells/day (Lee et al., 2022). However, these values were not significantly changed when the administration dose of heat‐killed TWK10 was reduced in this study (Table 2). Many studies have demonstrated that anti‐inflammatory responses induced by probiotics were dose‐dependent (Evrard et al., 2011; Ren et al., 2018; Sun et al., 2020). Therefore, we believe that the lack of significant improvement in the NLR and PLR values elevated by intense exercise in this study was due to the administration of an insufficient dose of heat‐killed TWK10.

Our previous studies have shown that the administration of different does (3 × 10^10^, 9 × 10^10^, and 3 × 10^11^ CFU/day) of viable TWK10 or heat‐killed TWK10 (3 × 10^11^ cells/day) reduced the serum lactate levels after exercise in humans (Huang et al., 2019; Lee et al., 2022). For parameters related to fatigue, we found that the administration of heat‐killed TWK10 (3 × 10^10^ cells/day) significantly reduced the production of serum lactate during exercise challenge (Figure 3b) in this study, which is consistent with our previous findings (Huang et al., 2019; Lee et al., 2022). At low and moderate exercise intensities, the predominant source of energy production is the fatty acid oxidation. When the exercise intensity increases, the source of energy production shifts from fatty acid oxidation to the glucose oxidation (Muscella et al., 2020). Glycolysis and lactate production increase proportional to the intensity of exercise (Philp et al., 2005). In our previous study, mice supplemented with viable TWK10 for 6 weeks upregulated the level of proteins required for fatty acid oxidation and transportation, such as peroxisomal acyl‐coenzyme A oxidase 2 (ACOX2), very long‐chain acyl‐CoA synthetase (S27A2), microsomal triglyceride transfer protein large subunit (MTP), and protein disulfide‐isomerase A4 (PDIA4) (Chen et al., 2020). Therefore, it is deduced that TWK10 provides the energy required for exercise mainly through promoting the oxidation of fatty acid rather than the breakdown of glucose, thereby reducing the production of lactic acid.

Ammonia, a waste product of nitrogenous compound metabolism formed during acute exercise, is considered one of the factors involved in the onset of exercise‐induced fatigue (Banister et al., 1983). The formation of ammonia during exercise involves the deamination of adenosine monophosphate and the catabolism of branched‐chain amino acids in skeletal muscles (Graham & MacLean, 1992). Ammonia production increases with exercise intensity and duration (Banister et al., 1983; Buono et al., 1984). In present study, the administration of heat‐killed TWK10 (3 × 10^10^ cells/day) reduced ammonia production after exercise and accelerated muscle recovery (Figure 3c), which is consistent with our previous findings (Huang et al., 2019; Lee et al., 2022). Therefore, administration of heat‐killed TWK10 for 6 weeks demonstrated potential anti‐fatigue benefits by reducing lactate and ammonia production during exercise.

Some probiotics and heat‐killed probiotics have shown the potentials to increase muscle weight and strength in animal and cell line models (Cheng et al., 2021; Lee, Hsu, et al., 2021; Lee, Kim, et al., 2021; Toda et al., 2020). Our previous studies demonstrated that administration of viable TWK10 increased muscle weight and strength in humans (Huang et al., 2019; Lee, Tu, et al., 2021), but administration of heat‐killed TWK10 did not increase muscle weight in humans (Lee et al., 2022). However, in this study, we demonstrated that the administration of heat‐killed TWK10 (3 × 10^10^ cells/day) for 6 weeks significantly improved muscle weight gain (Table 3) and muscle strength (Figure 2b) in humans. This contradiction between the results of the present study and those of our previous studies may be due to differences in the baselines of the subjects as well as the experimental designs. In addition, due to the limited samples collected in this human trial, cell‐based assays or animal study should be included to further investigate the mechanism of increased muscle weight gain and enhanced muscle strength exhibited by heat‐killed TWK10 in the future.

Consumption of probiotics has the potential to positively modify gut microbial community structures, which may be important to increase exercise performance in physical activity practitioners and athletes (Bressa et al., 2017; Clarke et al., 2014; Petersen et al., 2017; Scheiman et al., 2019), and the administration of heat‐killed probiotics show great potential for modulating the gut microbial composition and promoting health benefits (Asama et al., 2016; Berni Canani et al., 2017; Sawada et al., 2019). In our previous study, we have confirmed that heat‐killed TWK10 significantly increased the beta diversity of fecal microbiota. According to the gut microbial co‐occurrence network analysis, acetate‐ and butyrate‐producing bacteria, such as *Bifidobacteriaceae* (acetate), *Butyricicoccaceae* (butyrate), and *Ruminococcaceae* (butyrate), showed strong positive correlations with one another after administration of heat‐killed TWK10. In addition, subjects receiving heat‐killed TWK10 showed a significant increase in acetate levels (*p* < 0.05) in feces compared to the basal levels, and an increasing trend in propionate concentration was also detected in the heat‐killed TWK10 group (*p* = 0.0857) (Lee et al., 2022). Gut microbiota ferment non‐digestible dietary carbohydrates, which generate short‐chain fatty acids (SCFAs), such as acetate, propionate, butyrate, as the principal end products, and SCFAs play roles in skeletal muscle function and exercise capacity (Frampton et al., 2020). From these findings, we consider SCFAs play important roles in improving exercise performance and increasing muscle weight after administration of heat‐killed TWK10. Therefore, a multi‐omics approach should be employed to investigate the efficacy of the heat‐killed TWK10 in improving both phenotypic performance and gut microbiota in future studies.

## 5 CONCLUSIONS

In this study, we investigated whether administration of heat‐killed TWK10 exerted comparable effects to viable cells on exercise performance and health promotion in a double‐blind placebo‐controlled clinical trial. We confirmed that the administration of 30 billion cells of heat‐killed TWK10 for 6 weeks significantly reduced physical fatigue and improved exercise endurance capacity and handgrip strength in healthy humans without specific exercise training. Furthermore, consecutive administration of heat‐killed TWK10 significantly increased overall muscle weight in the absence of exercise intervention in the human body. By administration of heat‐killed TWK10, sufficient ergogenic effects comparable to 10 times higher doses of viable or heat‐killed cells were exerted in healthy adults without specific exercise training. Therefore, heat‐killed TWK10 could be considered as a potential ergogenic aid for improving aerobic endurance performance and as a potential effective nutritional supplement for muscle gain. Further investigation is required to address the functionality and understand the mechanisms underlying the beneficial effects of heat‐killed TWK10.

## AUTHOR CONTRIBUTIONS

The human clinical trial was done in the Sport Nutrition Laboratory, Graduate Institute of Sports Science, National Taiwan Sport University. Each author contributed the following: conceptualization, Chia‐Chia Lee, Jin‐Seng Lin, Chi‐Chang Huang, and Koichi Watanabe; formal analysis, Chia‐Chia Lee and Han‐Yin Hsu; investigation, Mon‐Chien Lee; resources, Jin‐Seng Lin and Chi‐Chang Huang; data curation, Mon‐Chien Lee and Han‐Yin Hsu; writing‐original draft preparation, Yi‐Chen Cheng; writing‐review and editing, Chia‐Chia Lee, Jin‐Seng Lin, and Koichi Watanabe; visualization, Yi‐Chen Cheng and Chia‐Chia Lee; supervision, Jin‐Seng Lin, Chi‐Chang Huang, and Koichi Watanabe; project administration, Chia‐Chia Lee and Han‐Yin Hsu. All authors have read and agreed to the published version of the manuscript, all persons designated as authors qualify for authorship, and all those who qualify for authorship are listed.

## FUNDING INFORMATION

This study received no external funding.

## CONFLICT OF INTEREST STATEMENT

Yi‐Chen Cheng, Chia‐Chia Lee, Han‐Yin Hsu, Jin‐Seng Lin, and Koichi Watanabe are employed by SYNBIO TECH INC. Mon‐Chien Lee and Chi‐Chang Huang declare no conflicts of interest.

## ETHICS STATEMENT

The study was conducted in accordance with the guidelines of the Declaration of Helsinki and was approved by the Institutional Review Board of Landseed International Hospital (Taoyuan, Taiwan; LSHIRB No. 19‐027‐A2).

## INFORMED CONSENT STATEMENT

Informed consent was obtained from all the subjects involved in the study. Before the trial, the researchers explained the trial process in detail, and the trial began after the subjects signed a consent form.

## Data Availability

The data presented in this study are available upon request from the corresponding author. The data are not publicly available because of the test sample patent and the subject's privacy and confidentiality.
